# Supplementary figures and images for: Household food security access and dietary diversity amidst COVID-19 pandemic in rural Nepal; an evidence from rapid assessment
Source: PLoS One. 2023 Nov 1;18(11):e0293514. doi: 10.1371/journal.pone.0293514 (PMC10619797; doi:10.1371/journal.pone.0293514)

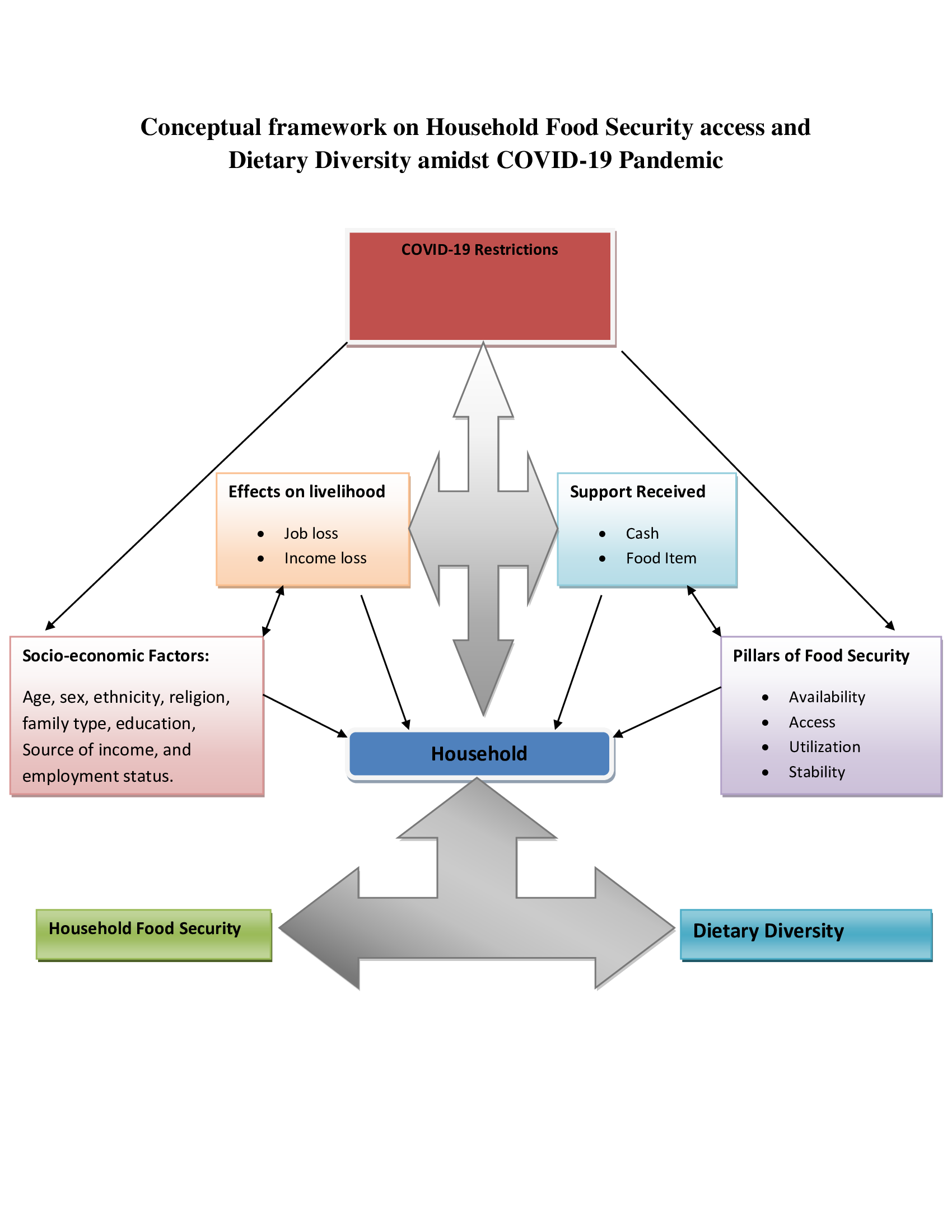

Supplement: S1 Fig — (TIF) [file pone.0293514.s001.tif]
