# Supplementary material for: Household food security access and dietary diversity amidst COVID-19 pandemic in rural Nepal; an evidence from rapid assessment
Source: PLoS One. 2023 Nov 1;18(11):e0293514. doi: 10.1371/journal.pone.0293514 (PMC10619797; doi:10.1371/journal.pone.0293514)
Supplement: Attachment — Submitted filename: Plosone@ Nepal.docx [file pone.0293514.s002.docx]

I appreciate all authors for this study. Although the study's research is quite unique, the authors should provide more related existing literature. Furthermore, the author didn`t clear properly why and this study is very relevant and significant and what is the policy implications? Please address this issue properly. The author(s) can also use a table to present a review of the literature. Determine the research gap in previous literature and how this work differs from previous efforts. Mention the gap in research. However, the methodology is clear and good.
The author can focus to create link how the results on this study fit in with the results from previous studies. The conclusion section is so weak, can provide summary of findings in this part. Author should discuss about research strength and limitations if have in this section. Lastly, I strongly suggest to give a paragraph about policy recommendations based on findings.
