# Supplementary material for: Household food security access and dietary diversity amidst COVID-19 pandemic in rural Nepal; an evidence from rapid assessment
Source: PLoS One. 2023 Nov 1;18(11):e0293514. doi: 10.1371/journal.pone.0293514 (PMC10619797; doi:10.1371/journal.pone.0293514)
Supplement: Attachment — Submitted filename: Response to reviewers, R2.docx [file pone.0293514.s004.docx]

September, 2023

The Academic Editor,

PLOS ONE

Subject: Resubmission of the research article entitled “Household Food Security access and Dietary Diversity amidst COVID-19 Pandemic in rural Nepal; an evidence from rapid assessment" (Manuscript ID: PONE-D-22-20978)

Dear Editor and Reviewers,

It is our great pleasure to submit our response to the reviewers on the comments made by reviewers and editor of our manuscript entitled "Household Food Security access and Dietary Diversity amidst COVID-19 Pandemic in rural Nepal; an evidence from rapid assessment” to PLOS ONE. Thank you for your concern regarding the quality of our manuscript. We really appreciate all the efforts of reviewers and editors as well as PLOS ONE journal team for providing us this opportunity to improve the quality of our research manuscript and reach wider audience worldwide.

We have addressed comments from both the reviewer and editor. We absolutely appreciate your concern and efforts in enhancing the quality of our manuscript. Here, we have highlighted our point-by-point response to the comments and the accompanying adjustments to the manuscript. We would be looking forward to your prompt decision regarding the publication status of our manuscript and our future collaboration with the PLOS ONE journal team.

We look forward to your kind consideration,

Thanking you,

Corresponding author

Dirghayu K.C.

Public Health Promotion and Development Organization

**Journals requirements**

Please review your reference list to ensure that it is complete and correct. If you have cited papers that have been retracted, please include the rationale for doing so in the manuscript text, or remove these references and replace them with relevant current references. Any changes to the reference list should be mentioned in the rebuttal letter that accompanies your revised manuscript. If you need to cite a retracted article, indicate the article’s retracted status in the References list and also include a citation and full reference for the retraction notice.

**Author’s response:** We have checked all the included reference article used in this manuscript from the reference list. We couldn’t find a retracted article in our reference list and were published online in health journals. However, we hereby want to notify PLOS ONE team that we have updated the whole reference section in our updated manuscript. To meet the journals requirement, the references have been updated which has been accompanied in our manuscript with track change and revised manuscript as well.

**Pont by point response to #Reviewer 1**

**Reviewer #1:** Page 43, Line 21: Please say 'COVID-19' pandemic for specificity.

**Author’s response:** Thank you for your suggestion. We have made the suggested changes.

**Reviewer #1:** Line 34: Think you mean to say Household Dietary Diversity Score (HDDS)...score is missing.

**Author’s response:** Thank you for your suggestion. We have made changes as per the reviewer’s suggestion.

**Reviewer #1:** Lines 34-36: Tenses used are not consistent - "whereas age and education are the predictors of HDDS", all other results are presented in past tense which makes sense. Also, when we say some is a predictor, there need some indication of directionality. Isn't the intent to say that age, ethnicity, educ, occupation are predictors of household food insecurity?

**Author’s response:** Thank you for your critical observation. We must have overlooked this during our initial revision. Tenses in the results section of abstract were adjusted as per the reviewer’s suggestion. Yes, we intend to present age, ethnicity, education, and occupation as the predictors of household food insecurity access. Amendment has been done in our revised manuscript following the suggestions of the reviewer.

**Reviewer #1:** Lines 105-107: Here and in the abstract to there is still a lot of use of causal language such as 'effects'. This is a cross-sectional and observational study. Any the analytic methods leveraged are not able to discuss any form of causal inference. Would temper language to just say that this is study examines the influence of the COVID-19 pandemic on household food security and household dietary diversity among rural communities in Nepal. Lines 109-113 again speak of impact.

**Author’s response:** Thank you for your constructive feedbacks. We have adjusted the use of language such as “effects” and “impacts” as per the reviewer’s suggestion.

**Reviewer #1:** Results section: The reporting of the results move back/ forth between discussing households as the unit of measurement to individual to families. Would encourage authors to provide some directionality when discussing associations or just state the ORs (lines 228-232).
Lines 238 + para that follows - Odds ratio do not reflect risk. The reflect odds. This section weaves in and out of reporting risk/odds.

**Author’s response:** Thank you for your comments and suggestions. As this study measures household food insecurity and dietary diversity among the households and the presented results are based on household as well as the participants. Some of our results needed to be presented based on the households characteristics whereas some needed to be presented based on the participants of the study. Hence, we have adjusted reporting of the results focusing on the households and individual participants interviewed wherever needed. We intended to present the socio-economic characteristics of our study participants in the line 228-232 based on the chi-square results obtained, their further association and ORs are discussed in the paragraph that follows whereby results of our bivariate and multi-variate analysis have been presented (line 239-248 of our tack change manuscript). We have adjusted our result section as per the reviewer’s suggestion and presented with reporting of odds wherever reviewer have pointed the need of reflecting odds.

**Reviewer #1:** Lines 289 - HFIAS is an indicator for food insecurity and HDDS is for dietary diversity, would simplify language to say thus say 'Despite the concerning high levels of food insecurity, household dietary diversity (HDDS>4) was low/medium for 63% of households in the study population and high for 36.8% of households.

**Author’s response:** Thank you for your suggestion and directionality. We have adopted and modified line 289 exactly as per the reviewer’s suggestion.

**Reviewer #1:** Would recommend strengthening the strength/limitations section.

**Author’s response:** Thank you for your constructive comments. A line has been added to strengthen our strength/limitation section as per the reviewer’s suggestion.

**Additional adjustments performed in the manuscript**

Apart from the comments made by editors, and reviewer, authors have made some changes in the manuscript to enhance its quality. Most of these additional changes are done to meet the formatting adjustments, addition of illustrative words, some basic corrections, and grammatical corrections in order to improve the quality of our final manuscript after publication. These amendments made by the authors can be observed in the track change file as listed below;

Abstracts

1. Line 21 "Covid-19" replaced with "COVID-19"; line 53 "Food insecurity" has been replaced with "food insecurity"

Introduction

2. Line 65 "United Nations Food Agriculture Organization" has been replaced with "Food Agriculture Organization (FAO) of the United Nations"; line 71 word "Furthermore" has been removed; line 83 "corona virus" replaced with "COVID-19"; line 94 "score" replaced with "scores"; "is" replaced with "are"; line 100 "Department of Health Services data 2019" replaced with "NDHS data 2016". Line 111 "effect" replaced with "influence".
3. Addition of space after before starting sentence and prior references wherever necessary.
4. Line 111-115, rational and objectives has been summarized in brief.

Methodology

5. Line 137, word "the" has been removed; line 140 the word "smallest" has been replaced with "lowest"; line 159 "HFS" replaced with "Household Food Insecurity Access Scale (HFIAS)"; "DDS" replaced with "HDDS"; line 164 addition of words "members of "; line 165 removed word "simply" line 166 addition of word "members of ".
6. Line 172, full form of the abbreviation "FAO-FANTA" has been provided; line 179, abbreviation "HDDS" has been presented in place of Household Dietary Diversity Score; Dietary Diversity Scores has been replaced with DDS in line no. 182,183,184; line 185, word "member of that particular household" has been added.

Results

7. Line 221, the word "household’s" has been replaced with "households"; word "to be" has been replaced with "being"; line 222, "nearly one-fourth being mildly food insecure" has been added; line 226, sociodemographic and COVID related characteristics has been removed; line 229-230 has been removed and added in line no 208-209; line 230-231 has been removed and added in line no 241-242; line 258-259 a line has been added to show the association of chronic disease and HFIAS.

Discussion

8. Line 276-280, discussion has been added in regard to our result association of chronic disease and HFIAS; Line 287-288, a sentence has been replaced to illustrate the potential impact of COVID restrictions on our study outcome.
9. Line 330-331, adjustment within the sentence has been made to better present the limitation of our study.
